# SERS detection of *Clostridium botulinum* neurotoxin serotypes A and B in buffer and serum: Towards the development of a biodefense test platform

**DOI:** 10.1016/j.acax.2018.100002

**Published:** 2018-12-21

**Authors:** China Y. Lim, Jennifer H. Granger, Marc D. Porter

**Affiliations:** a Department of Chemical Engineering, University of Utah, Salt Lake City, UT, 84112-5001, USA; b Nano Institute of Utah, University of Utah, Salt Lake City, UT, 84112-5001, USA; c Department of Chemistry, University of Utah, Salt Lake City, UT, 84112-5001, USA

**Keywords:** Botulinum neurotoxin detection, Biodefense, Bioterrorism, SERS, Immunoassay

## Abstract

Botulinum neurotoxins (BoNTs) are classified at a highest degree of threat in biodefense, due largely to their high lethality. With the growing risk of biowarfare, the shortcomings of the gold standard test for these neurotoxins, the mouse bioassay, have underscored the need to develop alternative diagnostic testing strategies. This paper reports on the detection of inactivated *Clostridium botulinum* neurotoxin serotype A (BoNT-A) and serotype B (BoNT-B), the two most important markers of botulism infection, by using a sandwich immunoassay, gold nanoparticle labels, and surface-enhanced Raman scattering (SERS) within the context of two threat scenarios. The first scenario mimics part of the analysis needed in response to a “white powder” threat by measuring both neurotoxins in phosphate-buffered saline (PBS), a biocompatible solvent often used to recover markers dispersed in a powdered matrix. The second scenario detects the two neurotoxins in spiked human serum to assess the clinical potential of the platform. The overall goal is to develop a test applicable to both scenarios in terms of projections of required levels of detection. We demonstrate the ability to measure BoNT-A and BoNT-B in PBS at a limit of detection (LoD) of 700 pg/mL (5 pM) and 84 pg/mL (0.6 pM), respectively, and in human serum at 1200 pg/mL (8 pM) and 91 pg/mL (0.6 pM), respectively, with a time to result under 24 h. The steps required to transform this platform into an onsite biodefense screening tool that can simultaneously and rapidly detect (<1 h) these and other agents are briefly discussed.

## Highlights

• Raman-based immunoassays can successfully detect botulism neurotoxins.
• Limits of detection for botulism neurotoxins A/B rival those of the mouse bioassay.
• Serum and liquid extracts are suitable sample matrices for the Raman assay.

## Graphical abstract

**Figure:**
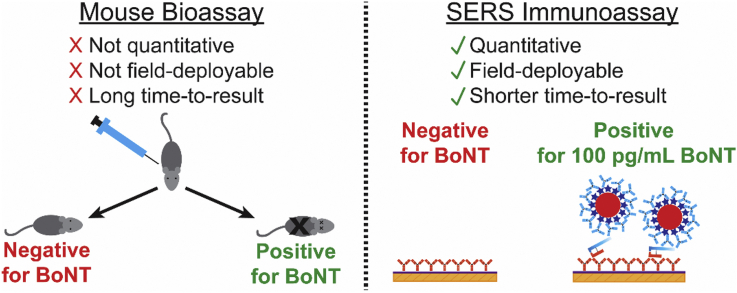

## 1 Introduction

Produced by *Clostridium botulinum*, botulinum neurotoxins (BoNTs) are among the most toxic substances known [1,2]. Exposure can result in potentially fatal muscle paralysis. Even with immediate intervention, recovery can require weeks to months of care [1,3]. The extremely high lethality of BoNTs has contributed to its ranking as a Category A pathogen by the U.S. Centers for Disease Control and Prevention (CDC), which parallels those by the European Union and Russia [4]. Other factors that led to this classification include the ease of BoNT dissemination, the potential for public panic, and the requirements for special public health practices post-exposure [3].

A simple calculation of the severity of BoNT from exposure by inhalation shows that a single gram of a dispersed BoNT aerosol, the most likely mode of dissemination, could kill up to 1 million people in a densely populated area [1,5]. This projection uses the LD_50_ (*i.e.,* the lethal dose leading to the death of 50% of an exposed population) for inhalation (10–13 ng/kg of body mass) and an adult weight of 70 kg. Other exposure pathways are also highly lethal. The LD_50_ for intravenous exposure is 1.3–2.1 ng/kg, and 1 μg/kg for ingestion [6]. These levels of lethality arise from a projection of studies on nonhuman primates with BoNT-A and BoNT-B, which are the most common and potent BoNTs [7], and underscore the need to develop rapid, low-level detection methods for BoNTs [1,3,8,9]. For applicability as a national security and public health tool, these tests should be capable of detecting BoNTs in a simple matrix (*e.g.,* a solvent often used to recover these markers when dispersed in a suspect “white-powder”) as well as in human serum [1,3,6,8,9].

**Fig. 1:**
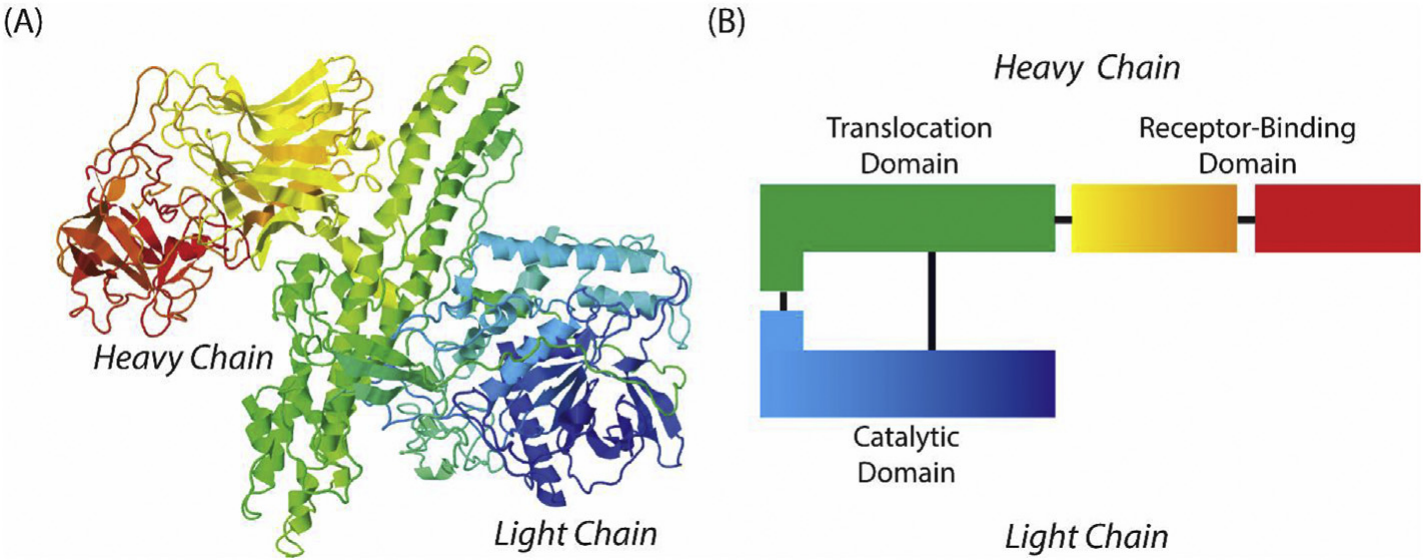
Botulinum toxin structure. (A) Crystal structure of BoNT A obtained from the Protein Data Base (PDB), PDB id: 3BTA. (B) Schematic representation of the crystal structure showing the heavy chain (translocation domain in green and receptor binding domains in red and orange/yellow) and the light chain (catalytic domain in blue). The color representation for (A) and (B) is the same. Fig. 1A was adapted with permission from Lacy et al. Nat. Struct. Biol. 1998, 5, 898–902. Fig. 1B was adapted with permission from The European Bioinformatics Institute (EBI) https://www.ebi.ac.uk/biomodels/content/model-of-the-month?year=2010&month=08, 2010.

Four (BoNT-A, BoNT-B, BoNT-E, and BoNT-F) of the seven BoNT serotypes can cause botulism in humans [6]. All BoNTs are 150 kDa zinc-dependent proteases composed of a heavy and a light chain coupled by a disulfide bridge [6]. The BoNT-A crystal structure [10] is shown in Fig. 1A, with a schematic representation in Fig. 1B. These toxins prevent nerve activation by cleavage of soluble N-ethylmaleimide-sensitive factor attachment protein receptors (SNAREs) responsible for synaptic vesicles docking with neurons [[11], [12], [13]]. The 100 kDa heavy chain translocates the toxin into a cell where the enzymatic 50 kDa light chain then blocks the release of neurotransmitters like acetylcholine by cleaving SNAREs. All BoNT serotypes work by similar mechanisms, each with a unique tertiary structure and amino acid sequence for a specific SNARE [12,14,15]. This paper focuses on detecting BoNT-A and BoNT-B, the two most prevalent of the neurotoxins and therefore the most likely serotypes to be used in a bioterrorism attack [16].

The timely and sensitive detection of BoNTs is central to the onsite screening of suspect biowarfare materials and the identification of infected patients. The most widely used test for BoNTs today is the mouse bioassay, which monitors mice for symptoms after inoculation with a suspect sample. This yes/no test has a limit of detection (LoD) of 20–30 pg/mL for BoNT-A and 10–20 pg/mL for BoNT-B, but can take up to 7 d to complete and cannot differentiate against other toxins that produce paralysis [1,17]. Due to the lengthy time to result, the diagnosis of botulism in humans relies on clinical suspicion (*i.e.,* patient history and symptoms) [3,18,19]. There are, however, several diseases with similar early stage symptoms, *e.g.*, Guillain-Barre syndrome, cerebrovascular accident, and myasthenia, that can complicate differential diagnosis [1].

As expected, a number of approaches have been developed to test for BoNTs that are aimed at overcoming the limitations of the mouse bioassay [18,[20], [21], [22]]. These include enzyme-linked immunosorbent assays (ELISAs) [[23], [24], [25], [26], [27], [28], [29], [30]], nucleic acid amplification tests (NAATs) [31], and a few other technologies [20,[32], [33], [34]], which can measure BoNTs at levels approaching that of the mouse bioassay, while also potentially overcoming the time, cost, and/or ease-of-use limitations. Serotype identification [35,36] is critical to choosing proper therapeutic treatment [37]. The work herein takes the first steps towards applying SERS, a plasmonics readout technology, that is potentially easier to implement with respect eventual field deployment over ELISA and molecular techniques.

**Fig. 2:**
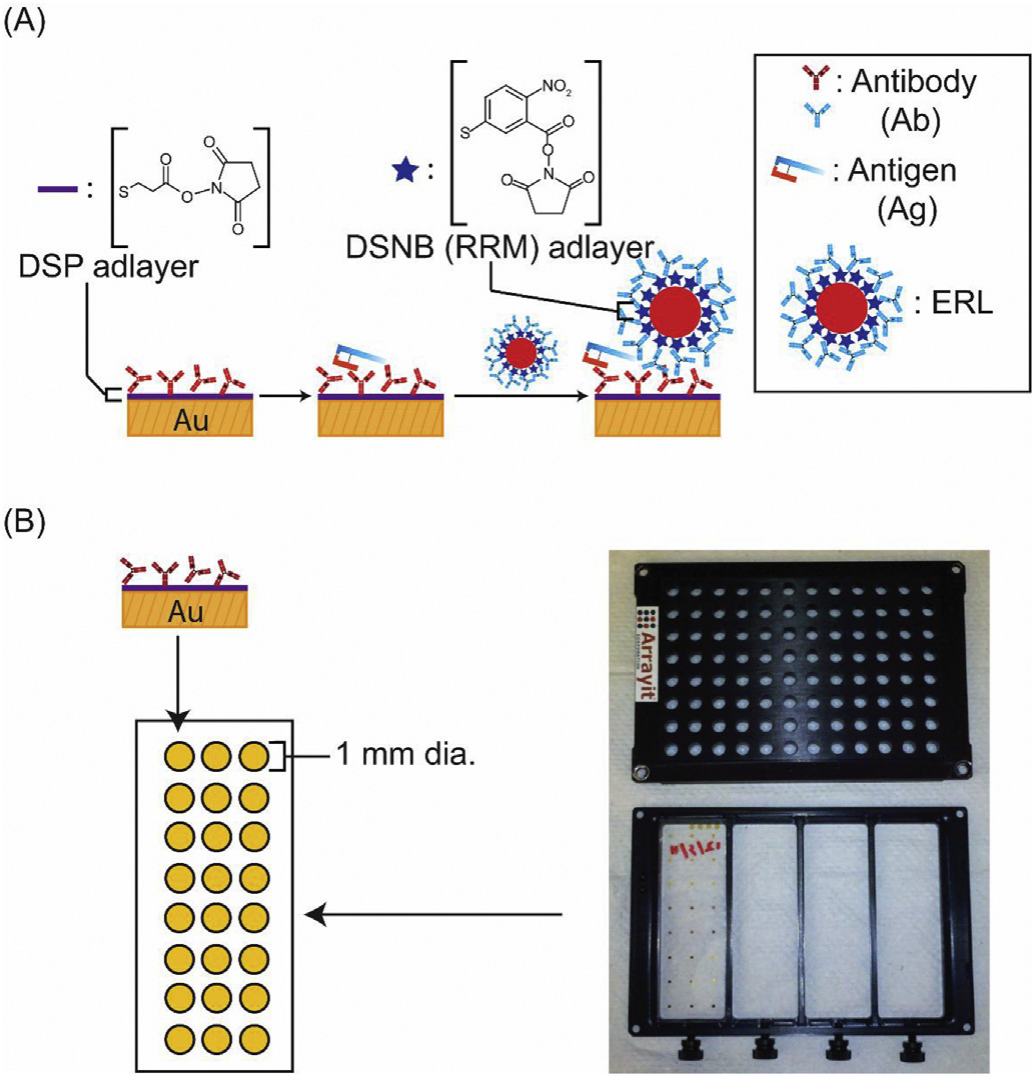
(A) Schematic of SERS-based sandwich immunoassay for BoNT-A and BoNT-B antigens. A gold address serves as the capture substrate after modification with dithiobis (succinimidyl propionate) (DSP) and BoNT-A or BoNT-B specific antibodies (Ab). After capture, the antigen (Ag) is labeled with extrinsic Raman labels (ERLs), consisting of AuNPs coated with a layer of a Raman reporter molecule, dithiobis (succinimidyl nitrobenzoate) (DSNB), and layer of BoNT-A or BoNT-B specific antibodies. (B) A 96-well Array-It^©^ plate defines gold addresses (1 mm dia.) on a glass microscope slide.

Our laboratory has focused on the development of highly sensitive immunoassays (Fig. 2A) for several diseases (*e.g.,* tuberculosis and prostate and pancreatic cancer) read out by surface-enhanced Raman scattering (SERS) [[38], [39], [40], [41], [42], [43], [44], [45], [46]]. This platform uses extrinsic Raman labels (ERLs), which are composed of gold nanoparticles coated with a Raman reporter molecule (RRM), tracer antibodies (Abs), and a capture substrate, which consists of a layer of antibodies adsorbed on a smooth gold film supported by a glass substrate. The amount of antigen (Ag) is indirectly quantified by the strength of the SERS signal from the RRM after ERLs tag captured Ags.

The use of SERS for readout has several attributes central to meeting this need. First, the spectral features of Raman scattering are much narrower than those of the electronic transitions operative in optical absorbance and fluorescence, which enables a higher level of spectral differentiation for different RRMs [[47], [48], [49]]. This capability is especially important in the development of a multi-marker pathogen platform, which can be realized by using a mixture of ERLs, each functionalized with a different RRM and tracer antibody [47,48]. Second, a multiplexed immunoassay with SERS can be analyzed by a single excitation source. This characteristic, along with advances in handheld Raman spectrometers, facilitates field deployment [[50], [51], [52]].

This paper describes the development and concept testing of SERS immunoassays for the low-level detection of BoNT-A (formalin-inactivated) and BoNT-B (light chain only). It begins with the results from optimizations and performance tests for measuring BoNT-A and BoNT-B in buffer and human serum, including identification of the most effective pairs of antibodies for antigen capture and labeling. The use of the two different sample matrices reflects the need to measure BoNT recovered from a “suspect” powder samples by means of a physiological buffer and in patient samples for medical diagnosis post-exposure. This is followed by sections that present the dose-response plots for detection of BoNT-A and BoNT-B spiked into both liquid matrices. The paper concludes with a brief projection of the steps required to move these measurements towards the development of a portable and rapid (<1 h) multi-marker test for active toxins and on integrating extraction procedures used when analyzing toxins in powdered matrices.

## 2 Materials and methods

**Bioreagents.** Polyclonal anti-botulinum neurotoxin type B (#AF5420, α-BoNT-B pAb) was purchased from R&D Systems. Polyclonal anti-botulinum neurotoxin type A [#730A, α-BoNT-A pAb (chicken IgY)], monoclonal anti-botulinum neurotoxin type A [#731A, α-BoNT-A mAb (mouse IgG)], botulinum neurotoxin type A toxoid [#133L, BoNT-A], and recombinant botulinum neurotoxin type B light chain [#620A, BoNT-B were obtained from List Biological Laboratories. All lyophilized bioreagents were reconstituted and stored per manufacturer recommendations.

**Other Reagents and Materials.** Dithiobis (succinimidyl propionate) (DSP, 95%), sulfuric acid (H_2_SO_4_) (ACS grade, 95.0–98.0%), methanol (CH_3_OH) (≥99.9%), and acetonitrile (ACN) were purchased from Sigma Aldrich; gold shot (99.999%) from Alfa Aesar; hydrogen peroxide (H_2_O_2_) (ACS grade, 30%), sodium chloride (NaCl), and Tween 20, from Fisher Scientific; 200-proof ethanol (EtOH) (ACS grade), from Pharmco-AAPER; modified Dulbecco's phosphate-buffered saline packs (PBS, 0.10 M, pH 7.8) and borate buffer packs (BB, 50 mM, pH 8.5), from Thermo Scientific; bovine serum albumin (BSA), from Jackson ImmunoResearch Laboratories, Inc.; gold nanoparticles (AuNPs, 60 nm), from Ted Pella, Inc., and human serum standards (Acusera), from Randox Laboratories. The synthesis of the Raman reporter molecule, 5-5′-dithiobis (succinimidyl-2-nitrobenzoate) (DSNB), has previously been described [38]. All chemicals were used as received. All aqueous solutions were prepared using water purified via a Barnstead polishing system to obtain ASTM type 1 water at 18.2 MΩ.

**Capture Substrate Preparation.** Before gold deposition, glass microscope slides (25 mm × 75 mm) were cleaned by piranha etch [3:1 sulfuric acid:hydrogen peroxide], rinsed with methanol, and dried with nitrogen. *Caution: Piranha etch reacts violently with most organic materials and must be used and handled with extreme care.* An aluminum mask was used to produce the 1 mm diameter gold addresses (3 × 8 array) on glass microscope slides by vapor deposition of a 200-nm coating of gold onto a 10-nm chromium adhesion layer. A DSP monolayer was formed on each gold address by immersion in an ethanolic DSP (0.10 mM) solution for 16 h. The slides were then thoroughly rinsed with ethanol, dried with nitrogen, and mounted onto the bottom of an Array-It^©^ platform (Arrayit Corporation, Fig. 2B) to form 96 distinct gold addresses in a 12 × 8 array.

Each of the following incubations used a sealing film (R&D systems) to reduce evaporation. First, a capture antibody layer was formed by the addition of 50 μL of 10 μg/mL α-BoNT-A mAb or α-BoNT-B pAb (PBS buffer, pH 7.4) to each well. After 3 h, the wells were rinsed three times by the addition of 100 μL of 10 mM PBS (pH 7.4) with 0.1% Tween-20 (PBS-T), followed by aspiration with a multi-channel pipet. The wells were then blocked for 1 h with 100 μL of 5% (w/v) nonfat milk in PBS for BoNT-A and 1.0% (w/v) BSA for BoNT-B in PBS to reduce nonspecific adsorption, and were then rinsed three times with PBS-T.

**Extrinsic Raman Label (ERL) Preparation.** 1.0 mL of the stock AuNP suspension (60 nm) was adjusted to pH ∼8.5 with 40 μL of 50 mM BB. The ERLs were then modified with 10 μL of 1.0 mM DSNB in ACN and mixed on a rotating plate for 3 h. A tracer antibody layer was then formed by the addition of 5.0 μg of α-BoNT-A mAb or 2.5 μg of α-BoNT-B pAb with rotation for 3 h. The next step added a 100 μL of 10% (w/v) BSA in 2 mM BB (pH 8.5) to the suspension for stability and to decrease nonspecific adsorption, followed by 30 min on a rotating plate. The ERLs were rinsed three times by centrifugation (2029*g*) for 10 min, and the supernatant was removed. The pellet was resuspended in 1.0 mL of 1% BSA in 2.0 mM BB for the first two rinses and 500 μL of 1% BSA in 2.0 mM BB for the final resuspension. The ionic strength of the suspension was adjusted by the addition of 50 μL of 1.7 M NaCl to obtain 150 mM NaCl. The ERL concentration of 4.9 × 10^10^ particles/mL was determined by UV/Vis spectrophotometry [53]. All the above steps were carried out at room temperature.

**Immunoassay Procedure.** All steps in the assay were carried out in a Biosafety Level 2 (BSL2) laboratory within a class III biosafety cabinet per guidelines from the Institutional Biosafety Committee of the University of Utah. Calibration standards were prepared by dilution from a stock solution of inactivated BoNT-A or BoNT-B in either PBS or directly in human serum. The standards and blanks were applied to the prepared capture surface (50 μL) and incubated for 3 h at room temperature; and the wells were then rinsed as previously described with 2.0 mM BB (150 mM sodium chloride, pH 8.5) with 0.1% Tween-20 (BB-T, 150 mM NaCl). The next step added as-prepared ERLs suspensions (50 μL) to each well for 16 h incubations at room temperature. Finally, the plate was rinsed using 2.0 mM BB (10 mM sodium chloride, pH 8.5) with 0.1% Tween-20 (BB-T, 10 mM NaCl) and allowed to fully dry (>1 h) at ambient temperatures. The slide was then removed from the Array-It^©^ assembly and analyzed via Raman spectroscopy.

**Raman Instrumentation.** Samples were analyzed using a DXR Raman microscope (Thermo Scientific) equipped with a 632.8 nm HeNe laser. The spectra were collected in an array fashion (6 × 6 array consisting of 36 spectra equally spaced at 50 μm intervals) using a 10 × objective (focal spot: 69 μm^2^), laser power of 5.0 ± 0.1 mW, 50 μm slit aperture, and 2 replicate scans with a 1.0 s integration time. Analysis included baseline correction before peak height determination of the symmetric nitro stretch [ν_s_ (NO_2_)] at 1336 cm^−1^ for DSNB, the RRM, for quantification of the bound antigen.

## 3 Results and discussion

**Fig. 3:**
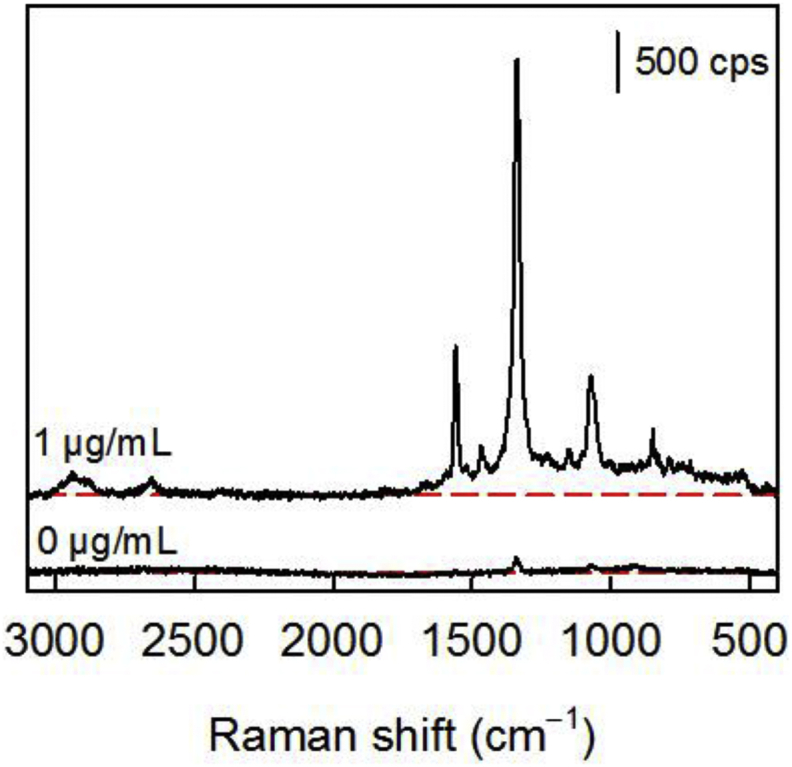
Raman spectra after a completed immunoassay (0 and 1 μg/mL BoNT-A in PBS) using mAbs as the capture antibody and pAbs as the tracer antibody. Both spectra are shown with a red dotted line representing the baseline response.

**SERS analysis of completed immunoassays.** To set the stage for the spectroscopic signatures used herein, SERS spectra from an assay for BoNT-A spiked in PBS (1 μg/mL) and a PBS blank are shown in Fig. 3. Characteristic Raman bands for the DSNB adlayer on the ERLs are clearly evident, including the symmetric nitro stretch [ν_s_(NO_2_)] at 1336 cm^−1^, the nitro bending mode [δ_s_(NO_2_)] at 848 cm^−1^, and aromatic ring stretching modes at 1556 and 1068 cm^−1^ [38,46]. The intensity of ν_s_(NO_2_) is used to indirectly quantify the antigen. The blank sample exhibits a small, but observable level of nonspecific adsorption. The studies below, portions of which are summarized in the Supplemental Information (SI), compare the SERS signals for 1 μg/mL BoNT (PBS) and a blank to determine a signal/blank ratio (R_S/B_), *i.e.,* a rough measure of the sensitivity of the assay.

**Antibody Selection for BoNT-A**. Based on qualitative indicators of performance reported in the specification information from a number of different commercial sources, two types of antibodies for BoNT-A were examined: monoclonal antibodies (mAbs) raised against the light chain of BoNT-A and polyclonal antibodies (pAb) raised against the heavy chain of BoNT-A. The epitope recognized by the F1-40 mAb [10] was mapped to the exposed loop on the light chain of BoNT-A. Much less is known about the epitopes targeted by the pAbs other than being raised against a fragment of the heavy chain of BoNT-A [54]. Both recognition elements were tested as potential capture/tracer antibody pairs (*i.e.,* mAb/mAb, pAb/mAb, pAb/pAb, and mAb/pAb).

**Fig. 4:**
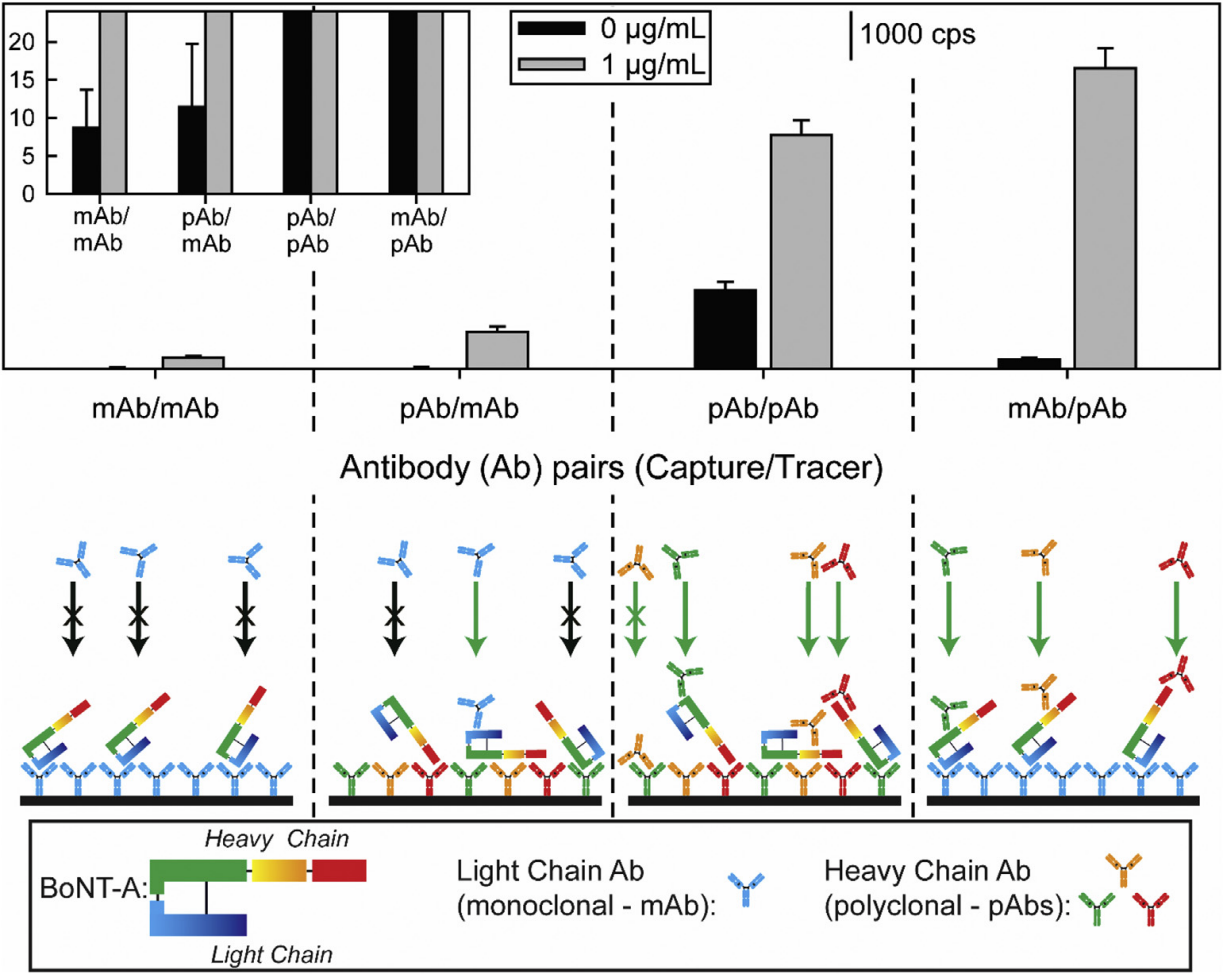
Upper panel: Antibody selection studies for BoNT-A for the SERS signal [νs (NO2)] for a completed assay for a PBS blank and PBS spiked with 1 μg/mL of BoNT-A with antibody pairs (capture/tracer): monoclonal antibodies (mAb) raised against the light chain of BoNT-A and polyclonal antibodies (pAb) raised against the heavy chain of BoNT-A. Lower panel: depiction of sandwich immunoassay without nanoparticle labels using various combinations of capture/tracer antibodies.

The top panel of Fig. 4 and Table 1 presents the results from this comparative study. As evident, the responses for BoNT-A are much stronger with the pAb as opposed to the mAb as the tracer antibody, but the pAb also increases the blank responses. Using the pAbs as the capture and tracer antibody results in the lowest R_S/B_ value due to a higher level of nonspecific adsorption. On the other hand, the pAb/mAb antibody pair produces the highest R_S/B_ value. However, the strength of the signal for the BoNT-A sample is much weaker than that for the mAb/pAb antibody pair. Thus, the mAb/pAb pair was used in all subsequent studies.

**Table 1:** Summary of the SERS signals using different combinations of capture/tracer antibodies for 1 μg/mL (S) and blank (B) PBS samples.^a^

Capture/Tracer Ab Combination	BoNT-A			BoNT-B		
	S	B	Ratio _S/B_	S	B	Ratio _S/B_
mAb/mAb	119	9	13	13	12	1
pAb/mAb	403	11	37	67	9	7
pAb/pAb	2561	858	3	1127	6	188
mAb/pAb	3292	103	32	325	21	15

a The noise level for the instrument for the same acquisition time is 18 ± 3 cps.

A basis for the differences in these data can be developed by considering the targeted specificity of the mAb for an epitope on the light chain of BoNT-A and that of the pAb for sites on its heavy chain while also recognizing the interplay of two other factors. First, pAbs recognize multiple epitopes on the antigen due to the production by multiple B cells. Second, mAbs target a single epitope with a comparatively stronger affinity reflective of their single cell lineage [55].

The use of the mAbs as both capture and tracer antibodies resulted in the lowest of the signals for these samples per the top panel in Fig. 4. This reflects the fact that only one epitope on BoNT-A is targeted by the mAb that was used for capture. This perspective is supported by the strong signal observed when using the mAb/pAb combination, which is depicted by the poor efficiency of the tracer mAb to tag captured Ag as shown in the lower panel of Fig. 4 [55,56]. The low signal level of the blank response is slightly above spectrometer noise. Using the pAb/mAb combination represents a small improvement in the ability to detect BoNT-A over that for the mAb/mAb combination. This suggests binding of BoNT-A through epitopes on its heavy chain to the immobilized layer of capture pAbs exposes a small portion of the epitope on the light chain of BoNT-A for tagging by the mAbs on the ERLs.

The responses for the BoNT-A spiked sample with the pAb/pAb and mAb/pAb combinations are much stronger that those in the two previous cases. In both instances, the ability to more efficiently label captured BoNT-A is realized through the use of the pAb as the tracer antibody, which we believe is reflective of greater steric accessibility to the heavy chain of the captured Ag. However, there is a higher level of nonspecific adsorption when using pAbs as the capture surface, which lowers the utility of the pAb/pAb combination over the mAb/pAb combination.

**Antibody Selection for BoNT-B**. Similar studies were carried out for BoNT-B with pAbs and mAbs, both of which in this case were raised against the light chain of BoNT-B. Using formalin-inactivated BoNT-B as the antigen produced little to no signal for any of the four different antibody pairings. We suspect that the lack of detectability is due to denaturation from the inactivation process. As a result, a light chain fragment of BoNT-B was used as a surrogate for the antigen in all data shown hereafter due to antibody availability. Studies to evaluate this assay format for inactivated but intact BoNT-B (*i.e.,* using means other than formalin inactivation) are being planned.

**Fig. 5:**
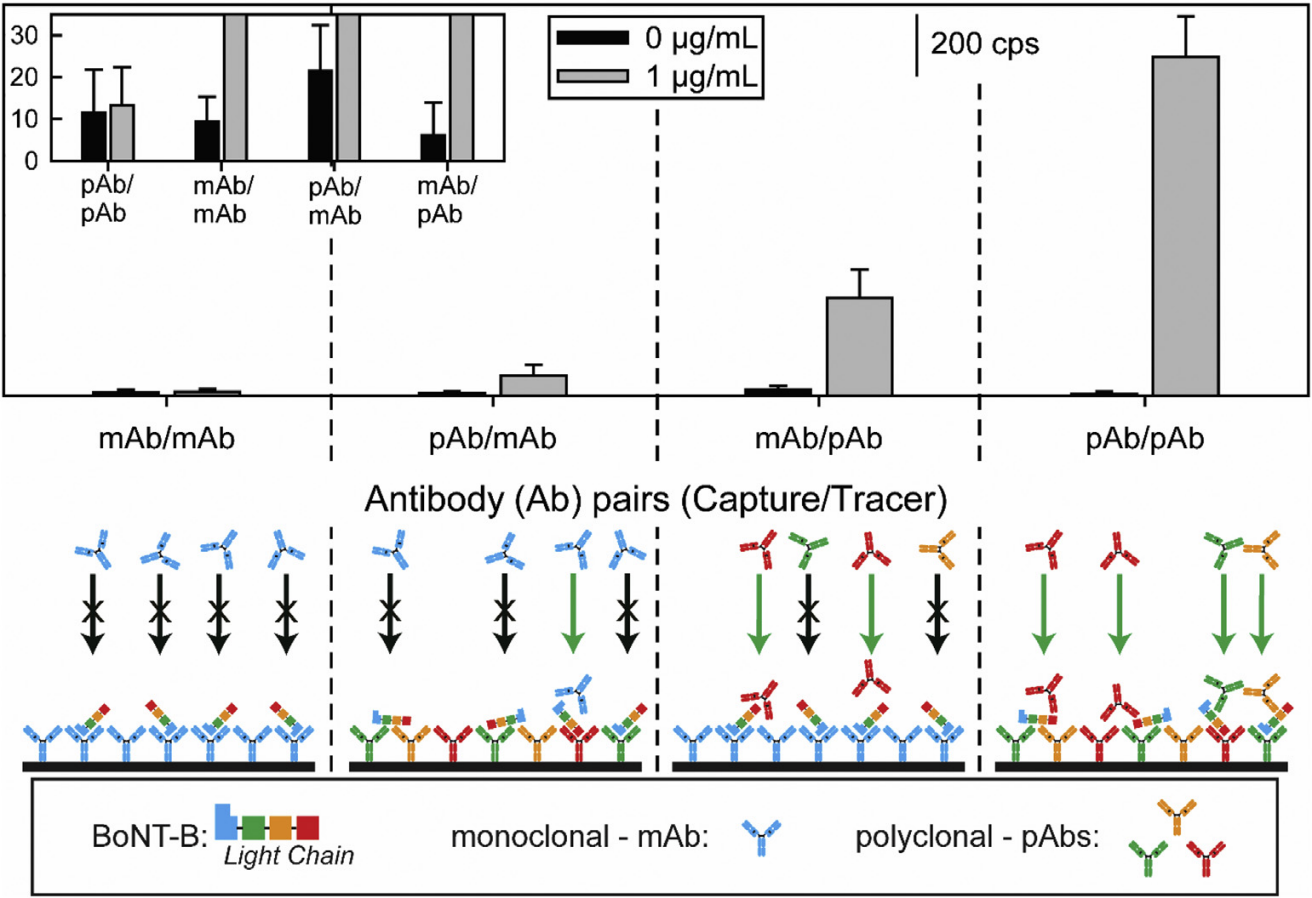
Upper panel: Antibody selection studies for BoNT-B for the SERS signal [ν_s_ (NO_2_)] for a completed assay for a PBS blank and PBS spiked with 1 μg/mL of BoNT-B with antibody pairs (capture/tracer): monoclonal antibodies (mAb) and polyclonal antibodies (pAb) raised against the light chain of BoNT-B. Lower panel: depiction of sandwich immunoassay without nanoparticle labels using various combinations of capture/tracer antibodies.

The results shown in Fig. 5 indicate that the use of pAbs as both the capture and tracer antibody performs the most effectively (R_S/B_ of 188). All other capture/tracer antibody combinations had much smaller R_S/B_ values (mAb/pAb: 15, mAb/mAb: 1, pAb/mAb: 7). Though the arguments for increased specificity with mAbs and increased binding efficiency with pAbs should still hold, the possible steric occlusion by use of the smaller, light chain of BoNT-B as the Ag alters some of the mechanistic perspective. As with BoNT-A, use of the mAbs for both capture and detection of BoNT-B gives a low signal for the 1 μg/mL sample, which is again attributed to steric occlusion due to the targeting of the same epitope. This condition is presented in the mAb/mAb capture/tagging depiction in Fig. 5. Transitioning to the pAb as a capture antibody slightly increases the signal for the BoNT-B sample due to a decrease in steric hindrance. Still, the use of mAbs as tracer antibody appears to be affected by steric occlusion. Using the mAbs as the capture antibody and the pAbs as the tracer antibody further increases the signal for the 1 μg/mL sample. Ultimately, increasing the level of steric accessibility of the light chain antigen appears to require use of the pAb for both the capture and detection of BoNT-B. Interestingly, and unlike the BoNT-A studies, the signal for the blank in this case is at levels on par with spectrometer noise.

**Assay Performance Tuning**. The next step determined the optimal amount of each antibody to use when coating the capture substrates and ERLs, as well as the composition of the diluent used for deposition. Although all antibody concentrations used in the coating processes are in theoretical excess (*i.e.,* >3 times the amount estimated to form a closest-packed monolayer), it is important to determine the absolute amounts needed for the most effective detection of antigen [57]. We also examined the addition of a nonionic surfactant, Tween 20, in the diluent (PBS buffer) employed in the preparation of the capture antibody coating. The tests using Tween 20 follow reports showing a reduction in protein adsorption on polystyrene (*e.g.*, the walls of 96-well microplates) by use of a nonionic surfactant [58].

**Fig. 6:**
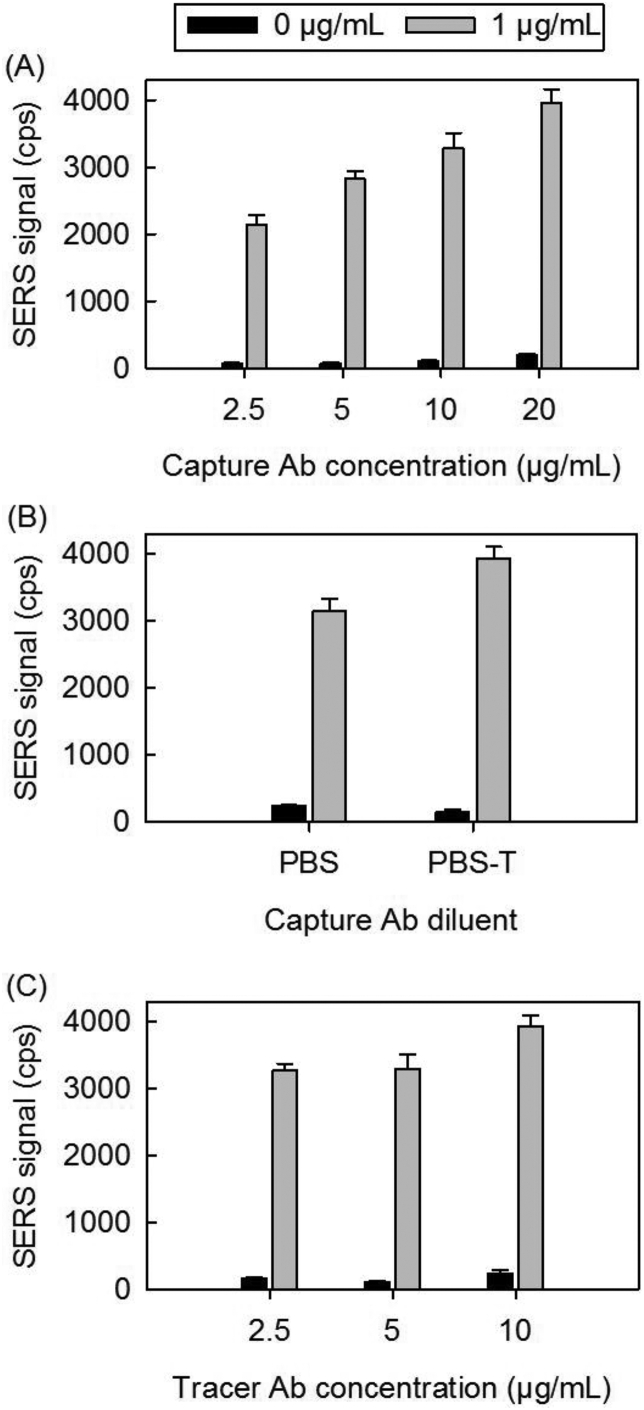
SERS signal [ν_s_ (NO_2_)] for 0 and 1 μg/mL BoNT-A for differing (A) capture antibody (Ab) concentrations; (B) capture Ab diluents; and (C) tracer Ab concentrations. The samples were prepared using the mAb as the capture antibody and pAbs as the tracer antibody.

The results obtained for the BoNT-A assays when using four different antibody concentrations in a PBS matrix to prepare the capture substrate are shown in Fig. 6A. In each experiment, the solution concentration of tracer antibody was fixed at 5 μg/mL. As evident, the responses for the samples and blanks increase with increasing capture antibody concentration. Interestingly, the R_S/B_ values are 33 ± 7, 40 ± 11, 32 ± 6, and 21 ± 3 for 2.5, 5, 10, and 20 μg/mL of capture antibody, respectively. Therefore, we opted to set the concentration of the capture antibody at 5 μg/mL when carrying out the more exacting performance assessments described later.

The results for assays using PBS and PBS with 0.1% Tween 20 (PBS-T) as diluents to prepare the capture antibody are summarized in Fig. 6B. These experiments used a capture antibody concentration of 10 μg/mL and tracer antibody concentration of 5 μg/mL, and gave R_S/B_ values of 13 ± 1 and 26 ± 4 for PBS and PBS-T, respectively. The addition of Tween 20 clearly reduces nonspecific adsorption, most likely by inhibiting hydrophobic interactions between the underlying DSP-based monolayer and the capture antibody [[59], [60], [61], [62]]. Experiments to investigate the effects of other additives are underway.

Finally, results for a completed BoNT-A assay with differing tracer antibody concentrations are shown in Fig. 6C, using a capture antibody concentration of 10 μg/mL in PBS. Similar to the trend observed with increasing capture antibody concentration, there is a slight increase in SERS signal intensity for both the sample and blank as the tracer antibody concentration increases to 10 μg/mL. However, there is no apparent dependence of the response at tracer antibody concentrations below 10 μg/mL. The blank sample gives a higher signal at a tracer antibody concentration of 2.5 μg/mL (162 cps) versus 5 μg/mL (103 cps). The 1 μg/mL sample gives comparable signals for both tracer antibody concentrations of 2.5 (3265 cps) and 5 μg/mL (3292 cps). The corresponding R_S/B_ values were calculated to be 20 ± 3, 32 ± 6, and 16 ± 3 for the tracer antibody concentrations of 2.5, 5, and 10 μg/mL, respectively. The optimum tracer antibody concentration with the largest R_S/B_ is obtained with 5 μg/mL tracer antibody.

Similar experiments were performed for the assay of BoNT-B, which yielded the highest R_S/B_ value at a capture antibody concentration of 5 μg/mL in PBS. With the tracer antibody concentration studies, the R_S/B_ values did not appreciably increase with increases in tracer antibody concentration; thus, a tracer antibody concentration of 2.5 μg/mL was chosen to reduce the consumption of antibody (*i.e.,* reduce costs). These data are shown in Figure S1.

**Fig. 7:**
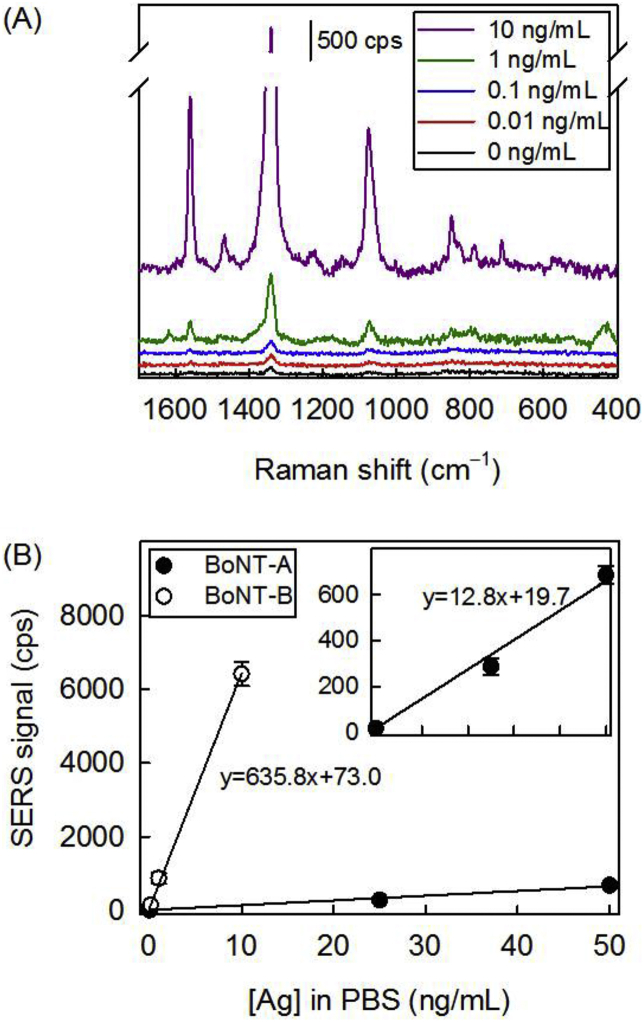
(A) SERS spectra of completed immunoassays showing trend of signal strength of ν_s_ (NO_2_) with BoNT-B concentration. (B) Dose response plots for BoNT-A and BoNT-B antigens by SERS signal [ν_s_ (NO_2_)] in PBS. The BoNT-A immunoassay was performed with the BoNT-A mAb (5 μg/mL) as the capture antibody and BoNT-A pAb (5 μg/mL) as the tracer antibody. The BoNT-B immunoassay was performed with the BoNT-B pAb (5 μg/mL) as the capture antibody and BoNT-B pAb (2.5 μg/mL) as the tracer antibody. Error bars represent the standard deviation of measurements of 3 samples.

**Dose Response Plots for BoNT-A and BoNT-B in PBS.** The guidance from these results were combined to construct dose-response plots for both neurotoxins in PBS as a means to assess the capabilities of the platform to test the analysis of the liquids used to extract toxins from “suspect” powder samples. Dilutions of BoNT-A and BoNT-B in PBS were prepared at concentrations up to 50 ng/mL. The resulting SERS spectra for BoNT-B and dose-response plots using the peak intensity of ν_s_ (NO_2_) relative to the BoNT-A or BoNT-B levels are shown in Fig. 7. The expected increase in the strength of ν_s_ (NO_2_) with increasing BoNT-B concentration is well evident in Fig. 7A. The spectra for the experiments with BoNT-A are presented in the Supplementary Data (Figure S2). Both sets of spectra were used to construct the dose-response plots in Fig. 7B.

These plots were used to estimate LoDs. The LoD, which correlates with the lowest concentration of captured antigen that can be detected at a confidence level of 95%, is calculated from the signal from the blank plus three times the standard deviation of the blank and the slope (*i.e.,* the sensitivity) from a least-squares linear fit to the dose-response plots. The LoDs in PBS are estimated to be 700 pg/mL (5 pM) for BoNT-A and 84 pg/mL (0.6 pM) for BoNT-B. To provide a perspective of the potential impact of this platform as a tool for pathogen detection, it is worthwhile to project an LoD that may be required when prepared for analysis of a toxin in the liquid generated from its extraction from a suspect white powder with a buffer like PBS [63]. Using the LD_50_ for intravenous exposure of BoNT-A (1.3 ng/kg) [6], which is the most demanding of the measurement scenarios, and an average adult weight of 70 kg, the corresponding toxic dose would be ∼90 ng. Given this, the amount relevant for detection after the extraction of toxin adsorbed onto a powder for aerosolization into 1 mL of PBS [64] translates to a concentration of ∼90 ng/mL or ∼600 pM. The LoDs estimated from the plots in Fig. 7B point to a detectability that is more than sufficient to meet this projection.

**Fig. 8:**
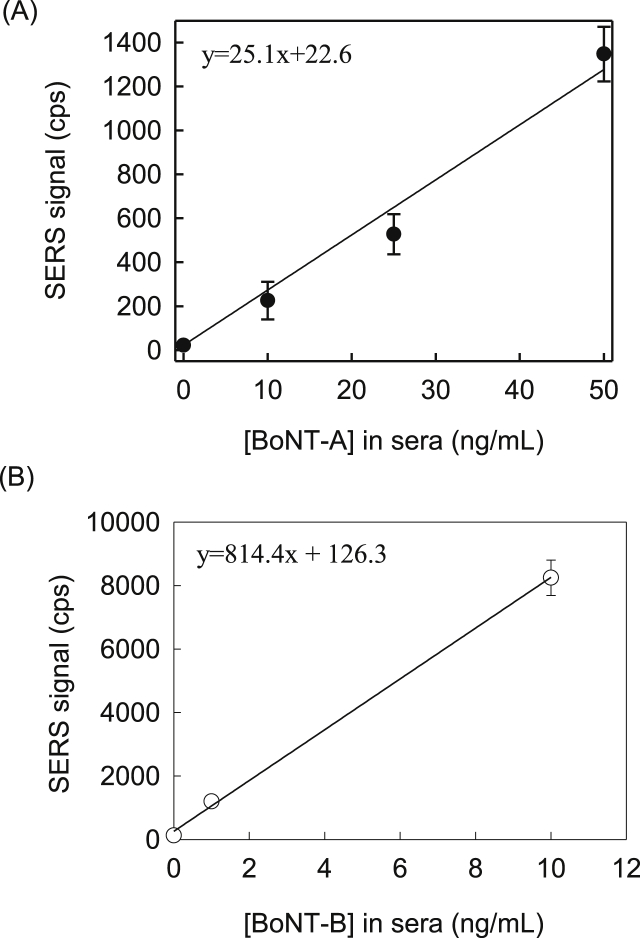
Dose response plots for (A) BoNT-A and (B) BoNT-B antigens by SERS signal [ν_s_ (NO_2_)] in human serum. Error bars represent average of 3 samples.

**Dose-Response Plots for BoNT-A and BoNT-B in Human Serum.** The dose-response plots for BoNT-A and BoNT-B spiked up to 25 ng/mL in human serum are shown in Fig. 8. The spectra used to generate each plot are shown in Figure S3. Analyses of these plots give LoDs of 1200 pg/mL (8 pM) for BoNT-A and 91 pg/mL (0.6 pM) for BoNT-B. These LoDs are close to those with PBS as a sample matrix. These results, as summarized in Table 2, indicate that there is little interference between any of the components in either assays, including that between the antigens and the serum matrix.

**Table 2:** Summary of LoDs for the detection of BoNT-A and BoNT-B in PBS and human serum using SERS-based immunoassays.

Antigen Matrix	LoD (pg/mL)	
	BoNT-A	BoNT-B
PBS	700	84
PBS (expected upon use of active toxin)	580	N/A
PBS (relevant LoD based on LD_50_)	6 × 10^5^ to 1 × 10^8^	
Human Serum	1200	91
Human Serum (expected with use of active toxin)	75	N/A
Human Serum (relevant LoD based on LD_50_)	20 to 200	

The relevant detection limit for BoNT in human serum, taking the most conservative toxic dose (*i.e.,* intravenous exposure with an LD_50_ of 1.3 ng/kg) and the blood volume and weight of an average adult (*i.e.,* 5 L and 70 kg), is ∼20 pg/mL (0.13 pM) [1,6,65,66]. With the more likely exposure for bioterrorism being inhalation (*i.e.,* LD_50_ of 10 ng/kg), the anticipated LoD for relevant detection of BoNTs, following similar calculations including blood volume, approaches that of 200 pg/mL 1.3 pM). Thus, the LoDs estimated for the detection of these two markers also appears sufficient for detection of BoNTs in human serum.

## 4 Conclusions

This paper detailed the development of a SERS immunoassay for the detection of two inactivated BoNT serotypes, BoNT-A and BoNT-B. After finely tuning the preparation of the capture substrate and ERLs, the approach gave LoDs for BoNT-A and BoNT-B spiked in PBS of 0.7 ng/mL and 84 pg/mL, respectively. Similar levels of detectability were achieved for the two markers directly from human serum with LoDs of 1200 pg/mL and 91 pg/mL for BoNT-A and BoNT- B, respectively. These LoDs demonstrate the potential of this approach for low-level detection of BoNTs in two relevant matrices.

The next steps will involve the use of active forms of BoNTs. Interestingly, past work has shown a reduction in the antigenicity of a toxin after exposure to formaldehyde by 5×  [[67], [68], [69]], which we anticipate will improve the LoDs by a comparable level [54,67,70,71]. The decrease in antigenicity is attributed conformational changes induced by inactivation [54,67,[70], [71], [72]]. As shown in Table 2, the reported LoDs are expected to be within relevant levels for both suspect sample screening and clinical diagnosis upon use of the active toxins.

These results, when combined with recent advancements in the development of portable Raman instrumentation, indicate that this strategy has the potential to be deployed in a mobile field laboratory as a tool for the detection of these, and possibly other, biowarfare agents. Work to further simplify and reduce the time required for the test procedure (<1 h), which will draw on our recent efforts using solid-phase extraction membranes [73] and a handheld Raman spectrometer for portable readout [74] and will integrate these methodologies with the extraction techniques used for suspect powdered samples, is underway.

## Conflicts of interest

None.

## Appendix A Supplementary data

BoNT Supplemental_Submitted BoNT Supplemental_Submitted
