# Supplementary material for: SERS detection of Clostridium botulinum neurotoxin serotypes A and B in buffer and serum: Towards the development of a biodefense test platform
Source: Anal Chim Acta X. 2018 Dec 21;1:100002. doi: 10.1016/j.acax.2018.100002 (PMC7587037; doi:10.1016/j.acax.2018.100002)
Supplement: BoNT Supplemental_Submitted [file mmc1.docx]

**Supplementary Data**

**SERS Detection of *Clostridium botulinum* Neurotoxin Serotypes A and B in Buffer and Serum: Towards the Development of a Biodefense Test Platform**

*China Y. Lim,^1^ Jennifer H. Granger,^2^* and Marc D. Porter^1-3^**

^1^Department of Chemical Engineering, ^2^Nano Institute of Utah, ^3^Department of Chemistry, University of Utah, Salt Lake City, UT, 84112-5001 USA

Table of Contents

1. *Figure S1: Bar graphs from measurements in the optimization studies for BoNT-B detection: (A) capture antibody concentration, (B) capture antibody diluent, and (C) tracer antibody concentration SI-2*
2. *Figure S2: Raman spectra for the immunoassay of BoNT-A in PBS SI-3*
3. *Figure S3: Raman spectra for immunoassay of BoNT-A and BoNT-B in serum SI-4*

**Figure S1.** SERS signal [ν_s_(NO_2_)] for blank and 10 ng/mL, 1 μg/mL, or 50 ng/mL BoNT-B for differing (A) capture Ab concentrations, (B) capture Ab diluents, and (C) tracer Ab concentrations. The samples were prepared using the pAb as both the capture and tracer antibody.

**Figure S2**. Representative Raman spectra of completed immunoassays in buffer showing trend of signal strength of ν_s_(NO_2_) at 1336 cm^-1^ with BoNT-A concentration.

**Figure S3**. Representative Raman spectra of completed immunoassays in serum. Plots show trend of signal strength of ν_s_(NO_2_) with (A) BoNT-A or (B) BoNT-B concentration.
